# Prediction of postoperative speech comprehension with the transcutaneous partially implantable bone conduction hearing system Osia®

**DOI:** 10.1007/s00106-023-01337-3

**Published:** 2023-10-09

**Authors:** Susan Arndt, Thomas Wesarg, Antje Aschendorff, Iva Speck, Thomas Hocke, Till Fabian Jakob, Ann-Kathrin Rauch

**Affiliations:** 1 Department of Otorhinolaryngology – Head and Neck Surgery, Medical Center, Faculty of Medicine, Albert Ludwig University Freiburg, Killianstr. 5, 79106 Freiburg, Germany; 2 Cochlear Deutschland GmbH & Co KG, Mailänder Straße 4 a, 30539 Hanover, Germany; 3 Klinik für Hals‑, Nasen- und Ohrenheilkunde, Universitätsklinikum Freiburg, Medizinische, Fakultät, Albert-Ludwigs-Universität Freiburg, Killianstr. 5, 79106 Freiburg, Germany

**Keywords:** Conductive hearing loss, Mixed hearing loss, Active transcutaneous bone conduction system, Speech comprehension, Bone-anchored hearing system

## Abstract

**Background:**

The active transcutaneous, partially implantable osseointegrated bone conduction system Cochlear™ Osia® (Cochlear, Sydney, Australia) has been approved for use in German-speaking countries since April 2021. The Osia is indicated for patients either having conductive (CHL) or mixed hearing loss (MHL) with an average bone conduction (BC) hearing loss of 55 dB HL or less, or having single-sided deafness (SSD).

**Objectives:**

The aim of this retrospective study was to investigate the prediction of postoperative speech recognition with Osia® and to evaluate the speech recognition of patients with MHL and in particular an aided dynamic range of less than 30 dB with Osia®.

**Materials and methods:**

Between 2017 and 2022, 29 adult patients were fitted with the Osia®, 10 patients (11 ears) with CHL and 19 patients (25 ears) with MHL. MHL was subdivided into two groups: MHL‑I with four-frequency pure-tone average in BC (BC-4PTA) ≥ 20 dB HL and < 40 dB HL (*n* = 15 patients; 20 ears) vs. MHL-II with BC-4PTA ≥ 40 dB HL (*n* = 4 patients; 5 ears). All patients tested a bone conduction hearing device on a softband preoperatively. Speech intelligibility in quiet was assessed preoperatively using the Freiburg monosyllabic test in unaided condition, with the trial BCHD preoperatively and with Osia® postoperatively with Osia®. The maximum word recognition score (mWRS) unaided and the word recognition score (WRS) with the test system at 65 dB SPL were correlated with the postoperative WRS with Osia® at 65 dB SPL.

**Results:**

Preoperative prediction of postoperative outcome with Osia® was better using the mWRS than by the WRS at 65 dB SPL with the test device on the softband. Postoperative WRS was most predictive for patients with CHL and less predictable for patients with mixed hearing loss with BC-4PTA ≥ 40 dB HL. For the test device on a softband, the achievable outcome tended to a minimum, with the mWRS tending to predict the realistically achievable outcome.

**Conclusion:**

Osia® can be used for the treatment of CHL and MHL within the indication limits. The average preoperative bone conduction hearing threshold also provides an approximate estimate of the postoperative WRS with Osia®, for which the most accurate prediction is obtained using the preoperative mWRS. Prediction accuracy decreases from a BC-4PTA of ≥ 40 dB HL.

The active partially implantable transcutaneous bone conduction system Cochlear™ Osia® (Cochlear, Sydney, Australia) has been approved in German-speaking countries since April 2021. The Osia is indicated for patients with conductive or mixed hearing loss (CHL/MHL) with an average bone conduction (BC) hearing loss (HL) of 55 dB HL or less in the frequencies of 0.5, 1, 2, and 4 kHz (four-frequency pure tone average, BC-PTA4) or with single-sided deafness (SSD; [12]). The advantage of transcutaneous compared with percutaneous BC systems is mainly the reduction in the incidence of skin inflammation and soft tissue reactions in the implant area. In addition, the cosmetic aspect needs to be considered. In persons with a short haircut, the percutaneous system can be particularly disadvantageous for esthetic reasons because of its visibility. For children, a transcutaneous solution is preferable with regard to swimming lessons and sporting leisure activities. Both systems bypass the disturbed sound transmission from the middle ear to the inner ear. Thus, in addition to the medical indication, the assessment of the audiological indication based on the BC hearing threshold is of central importance [13]. Another criterion for the indication and for estimating the expected success of the fitting is the maximum power output (MPO) of the BC system [13, 14]. The MPO represents the frequency-dependent course of the maximum power as a sound pressure level (dB SPL) or force level (dB FL).

Patients fitted with a BC system should have a dynamic range of 30–35 dB to be able to understand speech sufficiently [13]. If this required dynamic range is taken into account when determining the indication, the maximum possible average BC hearing threshold that can be provided is reduced to 40 dB HL (35 dB dynamic range) or 45 dB HL (30 dB dynamic range; [13]). Because of the steeper loudness growth, the minimum necessary target dynamic range can be reduced to 30 dB for BC implants [16]. The advantage of assessing treatment success with BC systems before implantation is that they can be tested by being worn on a soft band. However, the attenuation caused by the skin and hair, especially in the high-frequency range over 2 kHz, must be considered when adapting the test system, as this attenuation otherwise leads to a reduction in speech understanding of approximately 10 percentage points at 65 dB SPL [3]. One of the required conditions for a sufficient benefit of hearing aid fitting in Germany is a 20% improvement in speech understanding with the hearing aid at 65 dB SPL [17]. However, if unaided speech understanding at 65 dB SPL is not measurable, the maximum word recognition (mWRS) should be used as a prognostic tool for postoperative word recognition (WRS; [8, 9]).

The aim of this retrospective study was, therefore, to analyze the predictability of the postoperative WRS at 65 dB SPL with the Osia BC system by using the audiometric data available preoperatively (BC hearing threshold, unaided mWRS or the WRS at 65 dB SPL with BC test device on a soft band). Furthermore, we investigated whether the speech comprehension of patients with CHL achieved with Osia differs from that of patients with mixed HL and whether the AC threshold (air–bone gap, ABG) has an influence on the result. For this purpose, we analyzed the results of patients not reaching the required dynamic range of 30–35 dB on the basis of the BC-PTA4, i.e., who have an average BC threshold above 40 or 45 dB HL.

## Study design and methods

The present study was conducted with the approval of the Ethics Committee of the University of Freiburg (No. 21/1142) in accordance with national law and in accordance with the Declaration of Helsinki of 2013 (in the current, revised version; DRKS 00024640).

### Patients

A retrospective review included 29 adult patients (36 ears) with an Osia system (OSI100 or OSI200) implanted between 2017 and 2022. Nine of the patients (11 ears) had previously been enrolled in the multicenter CBAS5539 pilot study [10]. Another eight patients (12 ears) were included in the retrospective long-term analysis after Osia implantation, as published in 2022 [15]. Patient demographic and audiometric data are listed in Table 1.

**Table 1:** Patient demographic and audiological data

Demographic data						Audiological data									
						Preoperative unaided						Preoperative test on soft band		Postoperative Osia	
ID	Sex	Age (years)	Earlier study participation	Etiology	Side	Type of hearing loss	BC-PTA4 (dB HL) ipsilateral	AC-PTA4 (dB HL) ipsilateral	BC-PTA4 (dB HL) contra-lateral	AC-PTA4 (dB HL) contra-lateral	mWRS ipsilateral (%)	Test device	WRS@65 dB with test device (%)	WRS@65 dB with Osia (%)	Follow-up (mo)
1	M	37	1;2	Cholesteatoma bilateral, radical cavity right	Right left	CHL	18.7 12.5	61.2 33.7	–	–	100 100	Baha 5 Power Baha 5 Power	85 65	95 100	61
2	M	33	1;2	COM	Left	MHL‑I	25.0	60.0	Congenital deafness		90	Baha BP110	85	90	61
3	M	27	1;2	Atresia	Right	CHL	8.7	62.5	5.0	6.0	95	Baha BP110	90	90	61
4	W	61	1;2	Tympanosclerosis	Right	MHL-II	40.0	86.2	40.0	41.0	70	Baha BP110	30	80	61
5	M	18	1;2	Atresia (Nager syndrome)	Right left	MHL‑I	27.5 26.2	65.0 65.0	–	–	95 95	Baha BP110 Baha BP110	80 75	100 100	61
6	M	52	1;2	Cholesteatoma	Right	CHL	17.5	45.5	22.5	25.0	100	Baha BP110	80	95	58
7	W	77	1;2	Radical cavity	Right	MHL-II	47.5	92.5	–	–	65	Ponto 3 SP	45	60	58
8	W	30	1;2	Radical cavity	Right	MHL‑I	35.0	105.0	15.0	16.5	70	Baha 5 Power	50	85	58
9	M	39	1;2	Radical cavity	Left	CHL	8.7	66.7	6.0	7.7	95	Baha BP110	100	95	58
10	M	58	2	Radical cavity right, EC stenosis left, status post Baha left	Right left	MHL-II	41.2 45.0	69.2 55.6	–	–	90 90	Baha BP110 Baha BP110	50 70	65 80	50
11	W	64	2	Otitis externa	Right	MHL‑I	30.0	70.5	21.5	30.7	70	Baha 5 Power	65	70	50
12	M	40	2	Radical cavity right, status post bilateral Baha	Right left	MHL‑I	23.7 23.7	45.5 43.7	–	–	100 100	Baha 5 Power Baha 5 Power	80 80	95 95	51
13	W	43	2	Otosclerosis	Left	MHL-II	42.5	74.2	31.2	32.7	85	Baha 5 Power	35	80	45
14	W	69	2	Radical cavity	Left	CHL	18.7	51.75	18.0	20.7	95	Baha 5	95	90	39
15	M	52	2	Glomus tumor, EC obliteration	left	MHL‑I	22.5	77.5	21.2	25.0	80	Ponto 3 SP	80	80	39
16	W	57	2	Cholesteatoma bilateral radical cavity	Right left	MHL‑I	20.0 28.7	55.2 47.5	–	–	100 95	Ponto 3 SP Ponto 3 SP	85 85	95 95	38
17	M	59	2	Bilateral otitis externa	Right left	MHL‑I	31.2 33.7	55.0 88.7	–	–	95 80	Ponto 3 SP Ponto 3 SP	95 90	90 90	28
18	W	58	–	Radical cavity	Right	MHL‑I	26.2	60.5	19.5	27.0	100	Baha 5 Power	70	90	20
19	W	53	–	Cholesteatoma bilateral radical cavity	Right left	MHL‑I	35.0 30.0	53.5 41.2	–	–	100 100	Ponto 3 SP Ponto 3 SP	60 65	95 95	20
20	M	77	–	Radical cavity	Left	CHL	18.7	35	20.0	26.0	100	Baha 5	90	95	18
21	M	54	–	Cholesteatoma	Right	CHL	15.0	39.5	Deafness li, CI li		100	Baha 5	75	95	15
22	W	27	–	COM	Right	CHL	10.5	47.5	11.2	26.7	100	Baha 5 Power	90	95	13
23	M	55	–	EC stenosis	Left	MHL‑I	37.5	66.2	26.7	46.7	80	Baha 5 SP	60	85	11
24	M	60	–	EC stenosis	Right	MHL‑I	27.5	63	25.7	28.7	85	Baha 5 Power	60	80	6
25	W	36	–	Granulomatosis with polyangiitis	Left	MHL‑I	16.2	66.5	11.2	12.5	90	Baha 5 SP	65	75	5
26	M	46	–	Radical cavity	Left	MHL‑I	27.5	72.0	17.7	21.2	85	Ponto 3 SP	70	90	4
27	M	46	–	Cholesteatoma	Left	MHL‑I	33.7	78.0	28.7	30.5	75	Baha 5 Power	40	75	4
28	W	60	–	Tympanosclerosis	Left	MHL‑I	28.7	68.0	20.0	22.5	90	Ponto 3 SP	95	100	4
29	W	23	–	COM	Left	CHL	13.7	48.2	12.0	17.0	100	Baha 6 Max	100	95	1

*M* male, *W* female, *EC* ear canal, *COM* chronic otitis media, *Side* refers to implant side(s), *mo* months, *1* Mylanus et al. (2020), *2* Rauch et al. (2022), *CHL* conductive hearing loss PTA4 BC ≤ 20 dB HL, *MHL‑I* mixed hearing loss PTA4 BC 20–40 dB HL, *MHL-II* mixed hearing loss PTA4 BC ≥ 40 dB HL, *WRS@65* *dB* word recognition at 65 dB SPL, *mWRS* maximum word recognition possible at any SPL, *SP* SuperPower, *Baha* bone-anchored hearing aid

## Methods

### Audiological measurements and device programming

Data were collected as part of routine clinical practice for performance evaluation of the Osia implant. Device programming and audiological measurements were made in a soundproof booth (DIN EN ISO 8253). Preoperatively, the sound processors Baha BP110, Baha 5, Baha 5 SP and Baha 5 Power (Cochlear) and the sound processor Ponto 3 (Oticon Medical, Copenhagen, Denmark) were used as test devices, depending on the patient (Table 1). The prescriptive threshold-based method implemented in the fitting software was used to fit the sound processors to the soft band. Here, the frequency-specific gain requirement was determined based on the hearing thresholds measured in situ with the sound processor on the soft band. This pre-set was followed by fine tuning and, in the case of feedback, reduction of gain. The postoperative fitting of the Osia sound processor also included prescriptive determination of the frequency-specific gain requirement based on the in situ measured hearing thresholds, fine-tuning and gain reduction in the case of feedback.

### Hearing threshold determination

Pre- and postoperatively, the unaided thresholds in BC and AC were measured at the frequencies of 0.25, 0.5, 0.75, 1, 2, 3, 4, 6, and 8 kHz by means of headphones, the contralateral ear being masked with narrowband noise. In all patients, the PTA4 was determined in BC and AC preoperatively.

### Speech understanding in quiet

Preoperatively, unaided speech comprehension in quiet was determined using the Freiburg monosyllabic test with headphones (Beyerdynamic DT48 or Sennheiser HDA300) at various sound levels and the mWRS. One list per level was used. The maximum presentation level was 120 dB SPL. The measurements of the WRS were made at 65 dB SPL, preoperatively with the test device on the soft band and postoperatively with Osia, when the speech was presented from the front. To mask the contralateral ear, broadband noise at 70 dB SPL was presented via headphones. As this was a retrospective evaluation and because of organizational reasons and individual preferences, patients were given different test devices adapted to the respective BC-PTA4 thresholds for testing on the soft band (Table 1).

### Statistical analysis

After testing for normal distribution, group comparisons were made using mean comparisons by single-factor ANOVA and post hoc tests. According to the test for equality of variance, ANOVAs and Tukey post hoc tests were used for equal variances and Welch ANOVA and Games–Howell post hoc tests for unequal variances. The significance level was set at 0.05.

The Bonferroni correction was employed for multiple testing.

Simple linear regression was used to calculate the predictive accuracy of postoperative speech comprehension by using each of the two preoperative EV measures and the preoperative BC-PTA4. Goodness-of-fit or variance resolution was assessed according to the Cohen 1988 classification. The corrected *R*^2^ was used to avoid an overestimated effect for the variance increase *R*^2^.

The graphical correlation between the preoperative BC-PTA4 and the preoperative mWRS or the postoperative WRS was determined in each case by means of logistic regression according to the equation below. The Newton–Raphson method was used.

1 $$\mathrm{WRS}[{\%}]=100\frac {e^{(\beta _0 + \beta _1 \mathrm{PTA}4)}} {1+e^{(\beta _0 + \beta _1 \mathrm{PTA}4)}}$$

Analyses of variance and associated post hoc tests and linear regressions were performed using SPSS (version 27, IBM, Armonk, NY, USA) and nonlinear regressions using MATLAB (version 9.7, Mathworks, Natick, MA, USA).

The various results of the Freiburg monosyllable test were evaluated with regard to their significance levels according to Winkler and Holube [20].

## Results

Of the 29 patients included, seven had bilateral implants (36 ears in total). The patients were divided into three groups according to their mean BC hearing threshold: (1) CHL, (2A) MHL‑I, (2B) MHL-II. Of the 29 patients, 10 patients (11 ears) had a BC-PTA4 of < 20 dB HL and thus a CHL (1); 19 patients (25 ears) had a BC-PTA4 of > 20 dB HL and thus an MHL (2) and were then subdivided into a group with 20 dB HL ≤ BC-PTA4 < 40 dB HL (2A) (MHL‑I, *n* = 15 patients, 20 ears) vs. BC-PTA4 of ≥ 40 dB HL (2B, MHL-II, *n* = 4 patients, 5 ears). Furthermore, patients were further differentiated into three groups according to their ABG, as indicated by color markings in all figures (green: *n* = 5, ABG < 20 dB; blue: *n* = 19, 20 dB ≤ ABG < 40 dB; red: *n* = 12, ABG ≥ 40 dB).

### Preoperative audiometric results

The mean preoperative PTA4 on the implanted side for all patients was 26.4 ± 10.3 dB HL in BC and 61.6 ± 16.3 dB HL in AC.

The average hearing thresholds of the contralateral ear were 23.8 ± 9.8 dB HL (BC) and 37.8 ± 19.3 dB HL (AC).

**Fig. 1:**
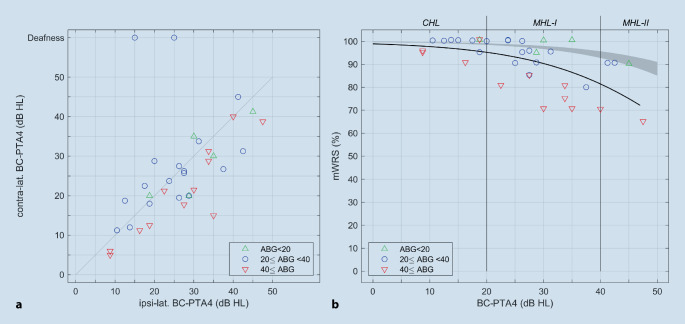
**a** Correlation between ipsilateral and contralateral averaged bone conduction hearing threshold (*BC-PTA4*). Values above the bisector indicate poorer contralateral bone conduction threshold. **b** Preoperative maximum word recognition score (*mWRS*) measured via air conduction as a function of BC-PTA4. The air–bone gap (*ABG*) is represented by different *colored symbols*. The *gray area* represents the mean mWRS as a function of the sensorineural hearing loss, taking into account the 95% confidence intervals for the parameters of a logical regression [4]. The *black line* indicates the mean mWRS; logistic regression according to Eq. 1

Figure 1a shows the relation of ipsilateral and contralateral BC hearing thresholds with respect to the side of the Osia implantation. The mean side-to-side difference of the BC-PTA4 was 6 dB. Of the 12 cases with an ipsilateral ABG ≥ 40 dB, 11 had a lower BC-PTA4 of the contralateral ear compared with the ipsilateral ear.

The mean differences of the thresholds between the ears for the two groups with lower AC threshold were below 2 dB, excluding the two cases with contralateral deafness.

The AC threshold was not significantly different between the three groups (Table 2). The preoperative mWRS was significantly different between the CHL and MHL‑I groups (Table 2). No significant differences were seen in preoperative mWRS between the CHL and MHL-II groups and between MHL‑I and II (Table 2). Preoperative WRS with the test device on the soft band was significantly better for CHL than for MHL-II (Table 2). In addition, the preoperative WRS with the test device on the soft band was significantly better for MHL‑I than for MHL-II (Table 2).

**Table 2:** Overview of results from variance analyses and post hoc mean comparisons of the hearing thresholds in the ear to be implanted or the implanted ear between the three groups

Variable investigated	ANOVA (*p*)	Group comparison: *p * and mean ± standard deviation (dB HL)		
		*CHL vs. MHL‑I*	*CHL vs. MHL-II*	*MHL‑I vs. MHL-II*
*Preoperative BC-PTA4*	*p* < 0.001	***p*** **<** 0.001 (***)^**a**^	***p*** **<** 0.001 (***)^**a**^	***p*** **<** 0.001 (***)^**a**^
		14.48 ± 3.91 vs. 28.69 ± 4.65	14.48 ± 3.91 vs. 43.25 ± 3.01	28.69 ± 4.65 vs. 43.25 ± 3.01
*Preoperative AC-PTA4*	*p* = 0.008	*p* = 0.051 (ns)^a^	***p*** **=** 0.009 (**)^**a**^	*p* = 0.265 (ns)^a^
		50.75 ± 12.09 vs. 64.06 ± 15.63	50.75 ± 12.09 vs. 75.57 ± 14.50	64.06 ± 15.63 vs. 75.57 ± 14.50
*Preoperative ABG*	*p* = 0.873	*p* = 0.984 (ns)^a^	*p* = 0.864 (ns)^a^	*p* = 0.903 (ns)^a^
		36.27 ± 13.64 vs. 35.38 ± 14.39	36.27 ± 13.64 vs. 32.32 ± 14.54	35.38 ± 14.39 vs. 32.32 ± 14.54
*Preoperative mWRS*	*p* = 0.005	***p*** **=** 0.007 (**)^**b**^	*p* = 0.081 (ns)^b^	*p* = 0.418 (ns)^b^
		97.73 ± 3.44 vs. 89.25 ± 10.42	97.73 ± 3.44 vs. 81.00 ± 12.45	89.25 ± 10.42 vs. 81.00 ± 12.45
*Preoperative WRS at65* *dB SPL with test on soft band*	*p* < 0.001	*p* = 0.085 (ns)^a^	***p*** **<** 0.001 (***)^**a**^	***p*** **=** 0.002 (**)^**a**^
		85.00 ± 12.45 vs. 73.00 ± 15.25	85.00 ± 12.45 vs. 46.00 ± 15.57	73.00 ± 15.25 vs. 46.00 ± 15.57
*Postoperative WRS at 65* *dB SPL with Osia*	*p* < 0.001	0.65 (ns)^a^	***p*** **<** 0.001 (***)^**a**^	***p*** **<** 0.001 (***)^**a**^
		92.73 ± 6.47 vs. 89.50 ± 8.26	92.73 ± 6.47 vs. 71.00 ± 8.94	89.50 ± 8.26 vs. 71.00 ± 8.94

**Bold** indicates statistical significance

*PTA4* four-frequency pure tone average, *BC* bone conduction, *AC* air conduction, *CHL* conductive hearing loss, *MHL‑I* mixed hearing loss (group I), *MHL-II* mixed hearing loss (group II), *(m)WRS* (maximum) word recognition score, *ns* not significant

^a^Tukey post hoc test

^b^Games–Howell post hoc test

### Preoperative mWRS as a function of average BC threshold

Figure 1b shows the preoperatively measured mWRS as a function of the averaged BC hearing threshold (BC-PTA4). The mWRS ranged from 65% to 100% for BC hearing thresholds up to 45 dB HL. The BC-PTA4 explained 37% of the variability found (*R*_Spearman_ = 0.61, *p* < 0.001). Cases with an ABG greater than 40 dB HL were significantly below the mean mWRS (shown as a black line as a function of BC-PTA4; sign test, *p* = 0.036). All cases with an ABG of less than 20 dB HL had an mWRS above this mean value.

The logistic regression according to Eq. 1 resulted in β_0_ = 4.50 ± 0.21 and β_1_ = −0.0756 ± 0.0061. The comparison with the mean mWRS of a patient group with pure sensorineural HL [5] reveals that, because of the measurement of the mWRS via AC, our patients show significantly lower values depending on the degree of sensorineural HL. Previously [4], values of β_0_ = 5.99 ± 0.08 and β_1_ = −0.0756 ± 0.0012 had been found. The larger confidence intervals for theβ parameters found in this study are attributable to the smaller number of cases.

### Postoperative audiometric results

**Fig. 2:**
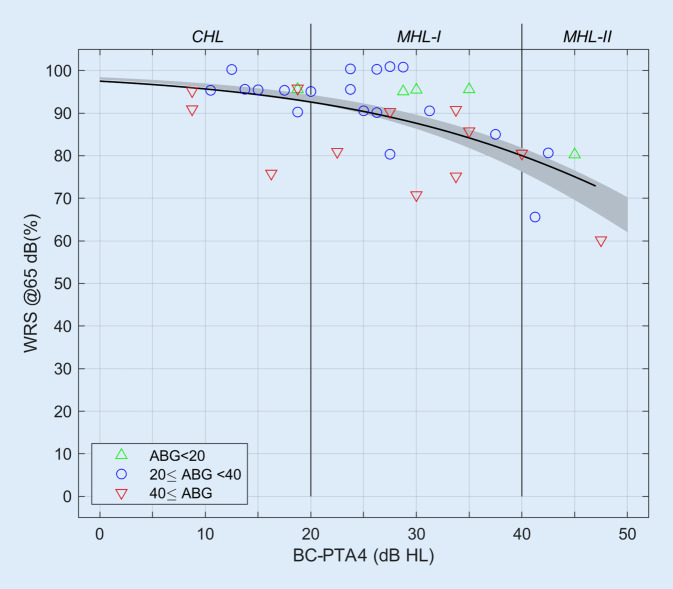
Postoperative word recognition score (*WRS*) achieved in the free field at 65 dB SPL with Osia as a function of the averaged bone conduction hearing threshold (*BC-PTA4*). The various air–bone gap (*ABG*) areas are represented by different *colored symbols*. The *gray area* represents the mean WRS with air conduction hearing aid as a function of the sensorineural hearing loss, taking into account the 95% confidence intervals for the parameters of a logical regression [5]. The *black line* represents the mean WRS with Osia; logistic regression according to Eq. 1

Figure 2 shows the WRS measured postoperatively as a function of the averaged BC hearing threshold. In the range of BC hearing thresholds up to 45 dB HL, the WRS achieved postoperatively was between 60% and 100%. However, the BC-PTA4 explained only 25% of the WRS variability found (*R*_Spearman_ = −0.51, *p* = 0.0016). In the cases with an ABG greater than 40 dB HL, the WRS was significantly lower than the mean WRS (shown as a black line; sign test, *p* = 0.036). In four out of five cases with an ABG of less than 20 dB HL, the WRS was greater than the mean WRS.

**Fig. 3:**
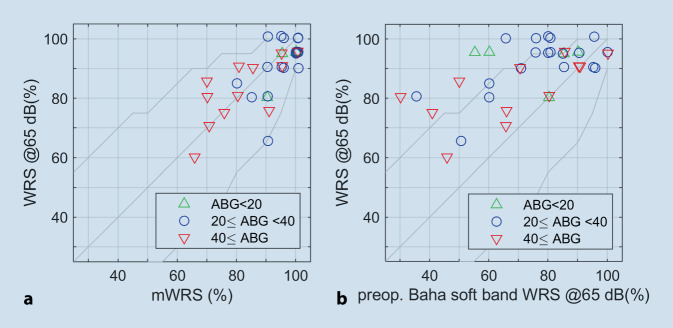
Relationships between pre- and postoperative word recognition score. **a** Word recognition score (*WRS*) achieved with the Osia above the maximum word recognition score (*mWRS*). **b** Relationship between WRS and preoperative WRS with the test device on the soft band. The air–bone gap (*ABG*) is represented by different *colored symbols*

Figure 3 shows the relationships between preoperative speech audiometric findings, mWRS (Fig. 3a) or WRS with the test device on the soft band (Fig. 3b), and postoperative WRS with Osia. Taking into account the small number of patients, the comparison of the two figures shows a tendency for the preoperative mWRS to be closer to the achievable fitting result than the WRS with preoperative testing with a test device on the soft band.

The logistic regression according to Eq. 1 gives β_0_ = 3.67 ± 0.17 and β_1_ = −0.0570 ± 0.0054. The comparison with a group of hearing aid users with exclusively sensorineural HL from a previous study ([5]; gray area) demonstrates that the mean fitting results with Osia are in the range of the 95% confidence interval for hearing aid users. The parameters were previously determined to be β_0_ = 3.98 ± 0.05 and β_1_ = −0.0661 ± 0.0008 [4].

### Prediction of postoperative audiometric results with Osia

#### Prediction based on preoperative BC-PTA4

Following the pooling of the results from all groups, the preoperative BC-PTA4 was able to predict postoperative WRS with Osia with a variance resolution of 31% (*R*^2^ = 0.310; *F*(1.34) = 16.709, *p* < 0.001; β = −0.570, *p* < 0.001). Linear regression for each of the individual groups was not significant (each *p *(ANOVA) ≥ 0.05). The linearity of the correlations was checked in advance by using a scatter plot. The linear relationship was not testable for the MHL-II group because of its small number.

#### Prediction based on preoperative WRS with test device on the soft band

On considering all groups, prediction of postoperative WRS with Osia using the preoperative WRS with test device on the soft band at 65 dB SPL was possible with a variance resolution of 36.3% (*R2* = 0.363; *F*(1.34) = 20.957, *p* (ANOVA) < 0.001; β = 0.338, *p* < 0.001). However, for the individual groups separately, a significant prediction could only be shown for the MHL‑I group with a variance resolution of 22.2% (*R*^2^ = 0.222; *F*(1.18) = 6.414, *p* (ANOVA) = 0.021; β = 0.288, *p* < 0.001, for CHL and MHL-II, each *p* (ANOVA) was ≥ 0.05).

#### Prediction based on preoperative mWRS

The preoperative mWRS of all patients significantly predicted the postoperative WRS with Osia with a variance resolution of 53% (*R*_2_ = 0.53; *F*(1.34) = 40.497, *p* (ANOVA) < 0.001; β = 0.716, *p* < 0.001). For each of the two groups, CHL and MHL‑I, the postoperative WRS with Osia could be estimated using the preoperative mWRS with a high variance resolution of 72.8% for the CHL group (*R*^2^ = 0.728; *F*(1.9) = 27.753, *p* (ANOVA) < 0.001; β = 1.635, *p* < 0.001) and high variance resolution of 61% for the MHL‑I group (*R*^2^ = 0.61; *F*(1.18) = 30.752, *p* (ANOVA) < 0.001; β = 0.654, *p* < 0.001). No prediction for MHL-II was possible based on preoperative mWRS (*p* (ANOVA) ≥ 0.05).

### Plot of postoperative WRS with Osia as a function of the preoperative test chosen

A mean preoperative mWRS of 91% was achieved or exceeded in 34 of the 36 cases (94%), within the accuracy of the Freiburg monosyllabic test [20]. The preoperative mean WRS of 73% with the test device was achieved or exceeded with Osia postoperatively in all cases. In none of the cases was a higher WRS than the mWRS observed in the preoperative testing with the soft band. Overall, the mean mWRS (94%) was less different from the mean postoperative WRS with Osia (88%) than the WRS achieved preoperatively with the test system (at 65 dB SPL).

## Discussion

Our results confirm that the Osia transcutaneous partially implantable BC system is a successful and safe alternative to percutaneous BC systems, as previously reported by other authors [1, 10, 15].

One of the aims of this retrospective study was to investigate the predictability of postoperative speech comprehension with the Osia, in order to be able to make a correct indication for implantation. This includes differentiation from alternative implants and the provision of adequate advice to patients about the potential achievable outcome with the Osia.

We were able to show that the patients achieved a level of WRS after Osia fitting, in line with current data from the hearing aid fitting of patients with exclusively sensorineural HL [2, 4]. Because of the signal presentation via free field for the mWRS, patients with mixed HL, especially those with a larger ABG, showed a lower mWRS preoperatively but were able to achieve this potential almost completely in contrast to conventional hearing aid users. The extent to which the sound level limitation of preoperative mWRS determination has an influence here remains unclear because of the retrospective design of the study.

The preoperative assessment of a successful fitting on the basis of the preoperatively recorded mWRS has been demonstrated several times by Hoppe et al. for patients with sensorineural HL without ABG [4–6]. For patients with CHL or MHL, there have been only two studies that examine the results after implantation of active middle ear implants and direct acoustic cochlear stimulators (DACS; [8, 9]). Müller et al. (2017) showed that the postoperative WRS at 65 dB SPL achieved with active middle ear implants is predominantly dependent on the coupling quality of the floating mass transducer. However, they make no mention of any influence of the ABG on the postoperative WRS. The mean deviation of two percentage points between the mWRS and the WRS with Osia, as found in our data, lies below the differences described for other implantable hearing systems [8, 9] and is most likely attributable to the standardized coupling and lower variability of the surgical approach compared with active middle ear implants.

Preoperative mWRS was better able to predict postoperative speech comprehension with Osia than preoperative WRS with a test device on a soft band, i.e., with higher variance resolution. If the skin attenuation of the transmission of the vibration was considered when the test device on the soft band was used, the results tended to show the minimum expected postoperative WRS. The mWRS, however, predicted the realistically expectable postoperative WRS at 65 dB SPL, and thus an overall higher estimate, which in our study was also still achieved or exceeded in 34 of 36 cases (94%) with Osia postoperatively.

Patients with a lower ABG had an improved postoperative WRS from preoperative mWRS with Osia compared to patients with a higher ABG. Thus, patients with an ABG of ≥ 40 dB HL achieved both a worse preoperative unaided mWRS and a significantly worse postoperative WRS with Osia at 65 dB SPL compared with patients having an ABG < 40 dB HL. In particular, the majority of patients with an ABG above 40 dB had a better contralateral ear. Based on these findings, which are contrary to the effect of improved postoperative WRS with Osia in patients with lower ABG, a better contralateral ear can be excluded as the reason for the observed effect. A potential cause could be the “listening” of the opposite ear via AC during the free-field measurement, i.e., with an ABG < 20 dB, the speech signal is transmitted both via the Osia with 65 dB SPL and directly via AC with < ~45 dB SPL (normal hearing: 100% WRS at 45 dB SPL). A contralateral transmission of the direct sound is also present with medium AC components, but at a lower level. Another possible explanation is a possible deprivation of the auditory pathway attributable to prolonged high-level conductive HL, especially with unusable conventional hearing aids prior to Osia fitting, such as in patients with auditory canal atresia [7, 21]. Our results show a negative dependence of speech comprehension with Osia on ABG and thus they differ from those reported by Mylanus et al. (1998) [11]. Snik et al. (2005) stated that BC hearing aids are superior to conventional AC hearing aids for conductive HL > 30 dB HL [19]. However, the differences in study design and the time of fitting between studies need to be considered. Mylanus et al. (1998) and Snik et al. (2005) refer to an analysis of an intra-individual comparison of conventional AC hearing aids and BC devices more than 25 years ago. The present paper presents current fitting results and their comparison with mean speech intelligibility from other studies/clinics in patient populations with pure sensorineural HL. A detailed clarification of the causes of the poorer results of Osia patients with an ABG > 40 dB shown in our work is not possible within the framework of this retrospective study. The dependencies of the postoperative WRS on the ABG, as indicated here, suggest the possibility of a preoperative estimation of the success of fitting with Osia. However, statistical confirmation of these observations should be performed in future prospective studies with an appropriate composition of the study population (and a larger number for the group of BC-PTA4 ≥ 40 dB HL).

Preoperative mWRS was able to predict postoperative WRS with Osia with high variance resolution and was more predictive than preoperative WRS with a test device on a soft band. Consideration of the different HL groups showed that preoperative mWRS was a good predictor of postoperative WRS with Osia for the CHL and MHL‑I groups, but not for the MHL-II group. The postoperative WRS of the MHL-II group had a wide range, with values between 60% and 80% WRS. Our results revealed, in particular, that patients with a BC-PTA4 ≥ 40 dB HL, who thus had a limited dynamic range of ≤ 30 dB [14], tended to achieve a WRS with Osia that differed only slightly from the the current cochlear implant indication of a WRS ≤ 60% WRS at 65 dB SPL in best aided condition (current German CI guideline) [18]. Thus, the indication limit of a maximum 55 dB HL with a BC-PTA4 should be considered individually and critically. Even though we studied only a limited number of patients with a BC-PTA4 ≥ 40 dB, our prediction models showed that the maximum possible treatable average BC hearing threshold should be reduced to 45 dB HL, rather 40 dB HL, in order to achieve sufficient aided speech recognition and to ensure tolerance regarding potential progression of HL. We investigated, in this study, the predictability of postoperative outcome with Osia as a function of preoperative BC-PTA4 and mWRS. Comparable studies with other BC systems are currently not available. We recommend prospective research on BC devices with the aim of determining the degree of postoperative benefit for patients in the borderline range of indication.

For patients with a BC-PTA4 ≥ 40 dB, an individual decision for the respective implantable hearing system (percutaneous superpower BC implant, active middle ear implant, cochlear implant) is particularly necessary and critical. Moreover, the progression and the etiology of the HL, the anatomical findings, and, above all, the age, the ability to undergo anesthesia, and the general condition of the patient must all be taken into account.

## Practical conclusion

- Osia can be used for the treatment of conductive and mixed hearing loss with an average, treatable, bone conduction hearing threshold of a maximum of 45 rather than 40 dB HL with good predictability of success.
- The preoperative word recognition (WRS) with a bone conduction test device on a soft band can be used for the lower estimation of the postoperative WRS with Osia.
- Both the mean preoperative bone conduction hearing threshold and the preoperative mWRS allow an estimation of the postoperative WRS to be made, which can be more accurately predicted using the preoperative maximum WRS.
- The present air–bone gap and the bone conduction hearing threshold are essential for the prediction of the speech comprehension that can be achieved postoperatively.
- The estimation of postoperative success has a high predictive accuracy for patients with conductive or mixed hearing loss with a bone conduction threshold of < 40 dB HL, whereas patients with a bone conduction threshold of ≥ 40 dB HL should be informed about the poorer predictability for their case and the potential non-achievement of the preoperative mWRS.

## Declarations

The present study was conducted with the approval of the Ethics Committee of the University of Freiburg (No. 21/1142) in accordance with national law and in accordance with the Declaration of Helsinki of 2013 (in the current, revised version; DRKS 00024640).

The supplement containing this article is not sponsored by industry.
